# Effect of Environmental Factors and an Emerging Parasitic Disease on Gut Microbiome of Wild Salmonid Fish

**DOI:** 10.1128/mSphere.00418-17

**Published:** 2017-12-20

**Authors:** Anti Vasemägi, Marko Visse, Veljo Kisand

**Affiliations:** a Department of Biology, University of Turku, Turku, Finland; b Institute of Veterinary Medicine and Animal Sciences, Estonian University of Life Sciences, Tartu, Estonia; c Institute of Ecology and Earth Sciences, University of Tartu, Tartu, Estonia; d Insitute of Technology, University of Tartu, Tartu, Estonia; University at Buffalo

**Keywords:** 16S rRNA gene fragment-based microbiome, host-gut microbial community interactions, proliferative kidney disease

## Abstract

The gastrointestinal tract (GIT) of fish supports a dynamic microbial ecosystem that is intimately linked to host nutrient acquisition, epithelial development, immune system priming, and disease prevention, and we are far from understanding the complex interactions among parasites, symbiotic gut bacteria, and host fitness. Here, we analyzed the effects of environmental factors and parasitic burdens on the microbial composition and diversity within the GIT of the brown trout (*Salmo trutta*). We focused on the emerging dangerous salmonid myxozoan parasite *Tetracapsuloides bryosalmonae*, which causes proliferative kidney disease in salmonid fish, to demonstrate the potential role of GIT micobiomes in the modulation of host-parasite relationships. The microbial diversity in the GIT displayed clear clustering according to the river of origin, while considerable variation was also found among fish from the same river. Environmental variables such as oxygen concentration, water temperature, and river morphometry strongly associated with both the river microbial community and the GIT microbiome, supporting the role of the environment in microbial assemblage and the relative insignificance of the host genotype and gender. Contrary to expectations, the parasite load exhibited a significant positive relationship with the richness of the GIT microbiome. Many operational taxonomic units (OTUs; *n* = 202) are more abundant in *T. bryosalmonae*-infected fish, suggesting that brown trout with large parasite burdens are prone to lose their GIT microbiome homeostasis. The OTUs with the strongest increase in infected trout are mostly nonpathogenic aquatic, anaerobic sediment/sludge, or ruminant bacteria. Our results underscore the significance of the interactions among parasitic disease, abiotic factors, and the GIT microbiome in disease etiology.

**IMPORTANCE** Cohabiting microorganisms play diverse and important roles in the biology of multicellular hosts, but their diversity and interactions with abiotic and biotic factors remain largely unsurveyed. Nevertheless, it is becoming increasingly clear that many properties of host phenotypes reflect contributions from the associated microbiome. We focus on a question of how parasites, the host genetic background, and abiotic factors influence the microbiome in salmonid hosts by using a host-parasite model consisting of wild brown trout (*Salmo trutta*) and the myxozoan *Tetracapsuloides bryosalmonae*, which causes widely distributed proliferative kidney disease. We show that parasite infection increases the frequency of bacteria from the surrounding river water community, reflecting impaired homeostasis in the fish gut. Our results also demonstrate the importance of abiotic environmental factors and host size in the assemblage of the gut microbiome of fish and the relative insignificance of the host genotype and gender.

## Abstract (precis)

Cohabiting microorganisms play diverse and important roles in the biology of multicellular hosts, but their diversity and interactions with abiotic and biotic factors remain largely unsurveyed. Nevertheless, it is becoming increasingly clear that many properties of host phenotypes reflect contributions from the associated microbiome. We focus on a question of how parasites, the host genetic background, and abiotic factors influence the microbiome in salmonid hosts by using a host-parasite model consisting of wild brown trout (*Salmo trutta*) and the myxozoan *Tetracapsuloides bryosalmonae*, which causes widely distributed proliferative kidney disease. We show that parasite infection increases the frequency of bacteria from the surrounding river water community, reflecting impaired homeostasis in the fish gut. Our results also demonstrate the importance of abiotic environmental factors and host size in the assemblage of the gut microbiome of fish and the relative insignificance of the host genotype and gender.

## INTRODUCTION

The interaction between microbiome diversity and host fitness has gained considerable attention among biologists in last decade, as host microbiomes, including those in the gastrointestinal tract (GIT), play critical roles in the host, including promoting health and providing “resistance” to opportunistic pathogens (1). In mammals, intestinal parasitic disease systems have been shown to significantly perturb GIT microbiomes (2–4) and the GIT has been suggested to play an important role in several extraintestinal diseases (5). Lower microbial diversity is also believed to be correlated with either a higher abundance of pathogenic bacteria or increased susceptibility to low-abundance opportunistic pathogens (6–8). In contrast to mammalian systems, we know very little about the interactions between the GIT microbiota and diseases in other organisms such as fish (9). Our limited knowledge, mostly based on farmed fish, suggests that fish-GIT microbiome interactions can be either beneficial or harmful to the host (10, 11). Bacterial pathogens may be present at low frequencies in healthy teleost microbiomes yet can emerge as pathogens under stressful circumstances (12, 13). Parasitic infections may also increase the risk of secondary bacterial diseases, as demonstrated in several experimental studies that show increased mortality rates of fish coinfected with parasites and bacteria (14). This synergistic effect has been explained by the elevated level of stress caused by parasites, which makes the host fish more vulnerable to secondary bacterial infections (15). Thus, an analysis of the GIT microbiota can be viewed as a valuable extension of the standard physiological markers of stress and health.

Proliferative kidney disease (PKD) is an emerging temperature-driven parasitic disease that occurs in both wild and farmed salmonid fish species in the northern hemisphere (reviewed in reference 16). Because a higher water temperature strongly magnifies disease symptoms and the mortality rate, PKD represents a serious threat for many salmonid populations (16–20). PKD is caused by the myxozoan parasite *Tetracapsuloides bryosalmonae*, which has a complex life cycle that includes two hosts, salmonid fish and sedentary freshwater bryozoans (commonly of the genera *Fredericella* and *Plumatella* [21, 22]). Fish are infected with parasite spores that develop in freshwater bryozoans (23). A mass release of *T. bryosalmonae* spores from bryozoans occurs in spring and early summer and results in synchronized fish infections that may occur as rapidly as within 10 min and reach 100% prevalence (23). After entering the salmonid host, the parasite multiplies in the blood and reaches the kidney, which is the primary organ where further development takes place (23, 24). The extrasporogonic stages of *T. bryosalmonae* spores undergo further proliferation that induces an inflammatory response and damages the kidney tissues in the host (17). Finally, the parasite migrates into the lumen of the kidney tubules, where spores able to infect bryozoans are excreted in the urine (25).

The clinical symptoms of PKD in salmonid fish depend on the temperature and include impaired excretion, kidney swelling, and anemia. Anemia decreases the performance of individual fish by lowering their aerobic scope and reducing their upper thermal tolerance (17). Consistent with an increased disease impact at rising temperatures, a massive PKD-driven kill of mountain whitefish (*Prosopium williamsoni*) has been recently reported in the Yellowstone River in Montana (26). During the onset of PKD, key cytokines that regulate the immune system are downregulated and the activity of granulocytes (components of the immune system) are depressed, thereby increasing the risk of contracting bacterial diseases (27). Thus, given the severe physiological consequences of the disease for the host (17, 28, 29), we hypothesized that PKD would also alter the composition of the GIT microbiota. Alternatively, the GIT microbiota may influence the status of the host immune system, which could influence the severity of the disease. For example, earlier work has shown that humans with low bacterial richness express a more pronounced inflammatory phenotype than individuals who have high bacterial richness (30). However, we do not know whether the GIT microbiota influences the progression of PKD in salmonids and, more generally, to what extent parasites affect the composition, function, and metabolic activity of the GIT microbiota in wild vertebrate populations.

The purpose of this study was to test if the abundance of *T. bryosalmonae* and the severity of PKD associate with the richness of bacteria in the GITs of juvenile trout collected from 10 genetically distinct but geographically close populations. On the basis of findings in other host-pathogen systems (31), we hypothesized that parr with severe PKD symptoms exhibit lower diversity/richness of GIT bacteria and are being colonized with more opportunistic/pathogenic bacteria. Furthermore, we predicted that the microbial diversity in the GIT system would show considerable intra- and interpopulation variations and a strong association with the ecological status/geomorphology and water temperature of the river.

## RESULTS

### Replication within and among river variations in operational taxonomic unit (OTU) abundance.

The variation between biological replicates of river water samples (*n* = 3) was not significant (type III sum of squares [SS] = 0.0309; pseudo-*F* = 0.4797; *P* = 0.875) in contrast to the variation between sampling sites (i.e., among rivers, SS = 4.0097, pseudo-*F* = 6.9137, and *P* < 0.001) on the basis of an analysis of variance (ANOVA)-like permutation test for distance-based redundancy analysis (dbRDA) [anova.cca() in the R vegan package]. The within-population variation among gut samples (*n* = 8 to 12 individuals per population) was marginally nonsignificant (SS = 0.527; pseudo-*F* = 1.4396, *P* = 0.062), while the variance between fish populations from different rivers was highly significant (SS = 9.566; pseudo-*F* = 2.9037, *P* < 0.001). The compositions of the microbial communities in the smaller subset of gut samples (*n* = 16 from seven rivers) that were analyzed between independent PCRs with the same DNA extract did not differ from each other (SS = 0.1813; pseudo-*F* = 0.6228, *P* = 0.922). This indicates that the technical replicates were similar while the variation between rivers based on the same small subset of samples remained highly significant (SS = 5.2231; pseudo-*F* = 2.9911, *P* < 0.001).

### Richness and alpha diversity of bacterial communities.

**FIG 1:**
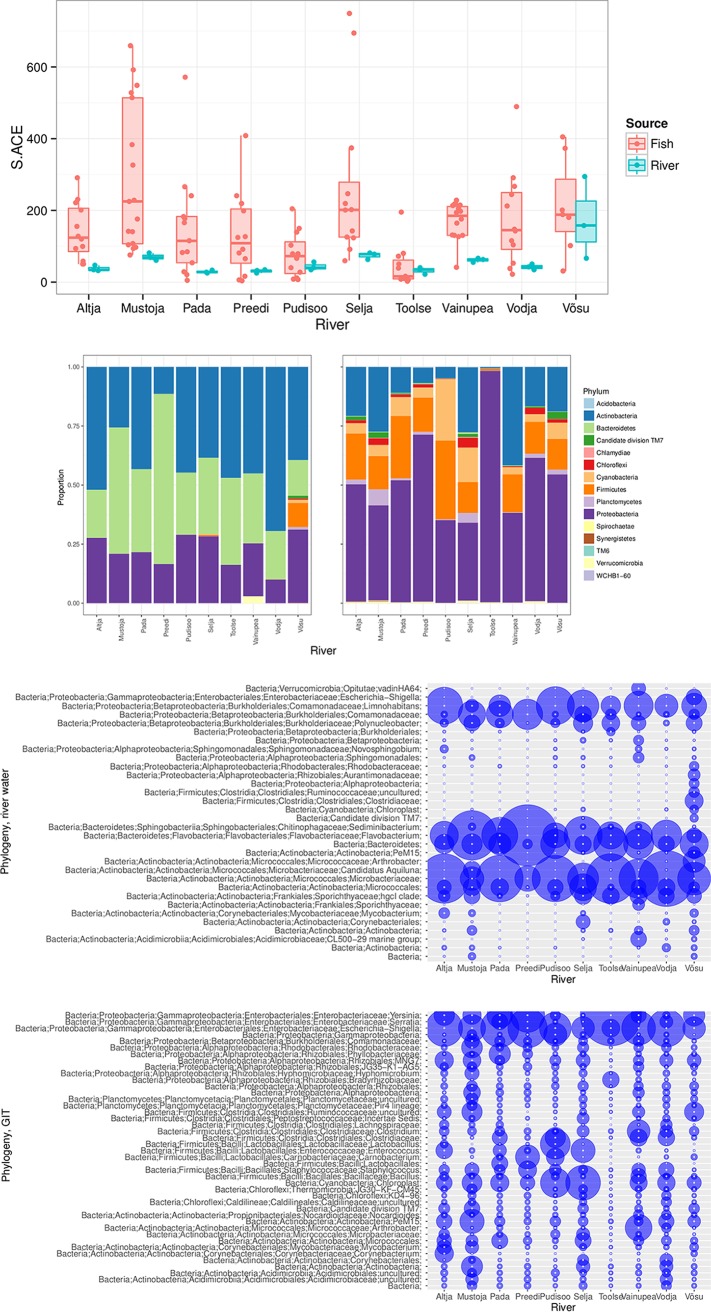
Shown are the numbers of predicted OTUs (S.ACE index, where S stands for species) in both river water samples and fish gut microbiomes grouped in a box plot with median 25 and 75% quartiles and outliers (top panel), the phylogenetic groups in river water samples (left) and gut samples (right) (second panel from the top), the abundance of the top OTUs in the guts of fish (*n* = 8 to 12) grouped by river (third panel from the top), and the abundance of the top OTUs in river water samples (three replicates pooled) (bottom panel).

The observed OTU count and richness estimated as the ACE index (32) (coefficient of variation, <3%) were similar in all samples. This suggests that all individual water and gut samples were sequenced exhaustively. This is further supported by a lack of singleton OTUs. Therefore, the ACE estimates were used as a realistic approximation of the total richness of the bacterial communities in the samples. In contrast to other water samples, we detected increased variation among three biological replicates from the River Võsu (Fig. 1, top). Nevertheless, the total richness in water samples was very similar among rivers and severalfold lower (median = 44) than that in the gut microbiome of juvenile brown trout (median = 130) (Fig. 1, top). The gut microbiome also varied significantly among rivers (permutational multivariate ANOVA [PERMANOVA]: pseudo-*F* = 3.9296; *R*^2^ = 0.23927; *P* = 0.001) and within rivers (pseudo-*F* = 1.4401; *R*^2^ = 0.00974; *P* = 0.039).

The microbial diversity within the water samples and the gut microbiome showed contrasting patterns at a high taxonomic level. In the water samples, the most dominant phyla were *Actinobacteria* and *Bacteriodetes*, followed by *Proteobacteria*, while the gut microbiome was dominated by *Proteobacteria*, *Firmicutes*, *Actinobacteria*, and *Cyanobacteria* (chloroplasts) (Fig. 1, second panel from the top). The most prominent genera in the gut included the genera *Shigella*, *Escherichia*, *Yersinia* (all enterobacteria), and *Bacillus* (Fig. 1, third panel from the top), while unidentified genera of *Microbacteriaceae* (*Actinobacteria*) and the genera *Flavobacterium* and *Limnohabitans* were dominant in the water samples (Fig. 1, bottom).

None of the OTUs were present in all of the gut samples, and only 14 were observed in >50% of the gut samples. The size of the “core community” as common OTUs in all river water samples was also low, as we found only 12 common OTUs in >50% of our samples. Two OTUs were observed in all of our water samples—*Flavobacterium* sp. (closest match, GenBank accession number EU801584) and an unidentified *Bacteroidetes* (closest match, GenBank accession number JF697404). In addition, two OTUs were observed in all but one water sample—an unidentified member of the family *Microbacteriaceae* (AJ575497) and an unidentified actinobacterium (AB599785).

### Beta diversity of bacterial communities.

The diversity of bacteria in the river water community differed from that in the gut microbiome (PERMANOVA: *F* = 27.1251, *R*^2^ = 0.134, *P* = 0.001). The variation in diversity among rivers was considerably lower than the variation of diversity in the GIT microbiomes of trout populations (Fig. S1). A robust pooling of OTUs according to their origins (GIT versus river water) revealed that 1,334 OTUs were unique to the GITs of fish while only 135 OTUs were unique to water samples. About 11% of the OTUs (*n* = 395) were found in both the GITs and river water samples (Fig. S2).

10.1128/mSphere.00418-17.1 FIG S1  Distribution of microbial communities in river water and GIT samples. PCo, principal component. Download FIG S1, TIF file, 0.1 MB. Copyright © 2017 Vasemägi et al. 2017 Vasemägi et al. This content is distributed under the terms of the Creative Commons Attribution 4.0 International license.

10.1128/mSphere.00418-17.2 FIG S2  Overlap of OTUs found in river water samples and GIT samples from *T. bryosalmonae*-positive and -negative fish. Download FIG S2, TIF file, 0.1 MB. Copyright © 2017 Vasemägi et al. 2017 Vasemägi et al. This content is distributed under the terms of the Creative Commons Attribution 4.0 International license.

**FIG 2:**
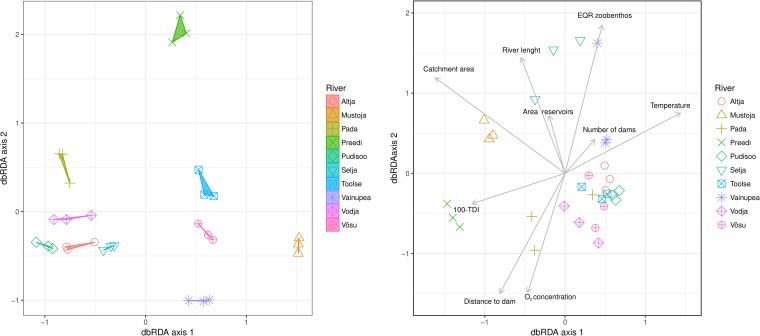
Bacterial community composition in river water samples analyzed by dbRDA. Sample scores are grouped according to the river on the left, and sample scores are plotted and related to various environmental and river morphometry data on the right. Arrows indicate how the metadata variables are related to ordination space. Only statistically significant metadata variables are shown.

Microbial communities varied among the rivers (Fig. 2, left), and this variation correlated with multiple abiotic variables, such as the level of oxygen (both concentration and saturation), water temperature, and river drainage morphometry (i.e., the size of the catchment area, river length, and the number of dams in the upstream water course) (Fig. 2, right; Table 1).

**TABLE 1:** Linear relationship of metadata variables with the ordination space of microbial diversity analyzed by dbRDA

Variable	River^a^			Fish^b^		
	Type III SS	*F*	Pr(>*F*)^c^	Type III SS	*F*	Pr(>*F*)
O_2_ concn	0.42004	6.692	0.001	1.157	3.1484	0.001
River length	0.18257	2.909	0.008	0.845	2.2988	0.001
Catchment	0.21453	3.418	0.004	0.864	2.3512	0.002
No. of dams	0.65827	10.488	0.001	1.041	2.8316	0.001
Area of reservoirs	0.57756	9.202	0.001	1.149	3.1265	0.001
Distance to dam	0.201	3.203	0.005	0.696	1.8949	0.007
Mean summer temp	0.72267	11.514	0.001	0.661	1.7982	0.012
100 − TDI	0.45178	7.198	0.001	0.743	2.022	0.002
EQR zoobenthos	0.6393	10.186	0.001	0.707	1.9241	0.008
Gender				0.482	1.2103	0.167
Fork length				1.007	2.5274	0.001
Mass				1.161	2.9146	0.001
Kidney ratio				0.537	1.3469	0.086
Hematocrit				0.384	0.9626	0.488
Relative infection				0.737	1.8489	0.008

a Relationships in river water microbial community.

b Relationships in gut content microbial community.

c Pr(>*F*), observed significance levels for the *F* statistic.

**FIG 3:**
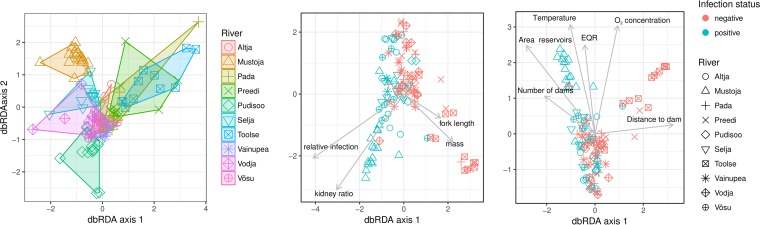
The graph on the left shows variations of microbiome diversity in the guts of fish plotted as dbRDA sample scores. The graph in the middle shows the relationships of the gut microbiome to measured environmental variables (for details see Table S1), and the symbols are sample scores of gut samples. The graph on the right shows the relationships between the gut microbiome and traits of fish. Arrows indicate how statistically significant variables are related to dbRDA axes.

The diversity of the gut microbiome was highly variable among fish taken from the same river but displayed clear clustering according to the population of origin (Fig. 3, left). The GIT microbiomes in the Mustoja and Selja rivers were the most divergent from those in the other rivers. Both of these rivers have several large man-made reservoirs upstream of the sampling sites. As with the microbial community in river water, measured environmental variables strongly associate with the bacterial composition within the fish gut microbiome (Table 1; Fig. 3, middle). In addition, both fish length and mass showed significant relationships with the gut microbiome but not with gender (Table 1; Fig. 3, right). Among PKD-related traits, only the relative parasite load exhibited a significant relationship to the gut microbiome (Table 1; Fig. 3, right). An additional test of the effect of the parasite load on microbiome richness in the GIT indicated that microbiome richness increases with the parasite load (SS = 49.89114, *t* = 2.207981, *P* = 0.0297); however, there are some differences between rivers (see Fig. 2). Although the geographic distance and genetic divergence between fish populations showed a strong positive correlation (Mantel test *r*_xy_ = 0.70, *P* = 0.017), no significant relationships between geographic distance and the (Bray-Curtis) dissimilarity between GIT microbiomes between rivers was found (Mantel test *r*_xy_ = 0.016, *P* = 0.42). Likewise, neither the host population genetic diversity (*A*_r_) nor the geographic distance correlated with the GIT microbiome (Shannon) diversity index (Spearman’s rank correlation coefficient = 0.076, *P* = 0.341).

10.1128/mSphere.00418-17.3 TABLE S1  Ecological status and morphometry of rivers studied. Download TABLE S1, DOC file, 0.04 MB. Copyright © 2017 Vasemägi et al. 2017 Vasemägi et al. This content is distributed under the terms of the Creative Commons Attribution 4.0 International license.

### Bacteria characteristic of *T. bryosalmonae*-infected fish.

**FIG 4:**
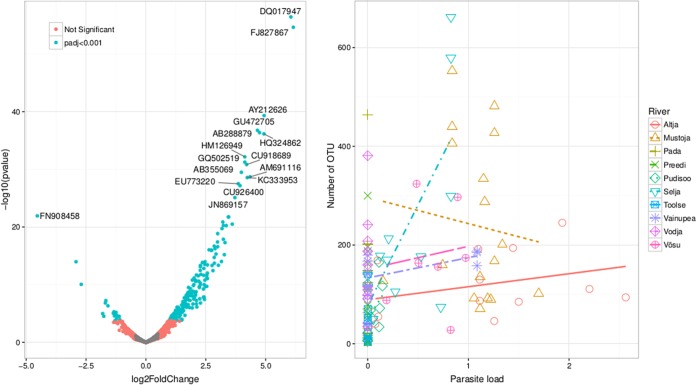
The graph on the left shows the OTUs (NCBI accession numbers of the closest matches) overrepresented in abundance in the GITs of *T. bryosalmonae*-positive and -free trout (log_2_ fold change, >0). The OTUs less abundant in *T. bryosalmonae*-positive trout than in *T. bryosalmonae*-free fish have a log_2_ fold change of <0. The graph on the right shows relationships between parasite loads (square-root-transformed relative parasite DNA amounts) and richness (number of OTUs per sample) modeled by using the mixed generalized linear model. The more complex model with interaction with richness × river presented is marginally better than the model without the interaction (AICs, 1,509.4 and 1,502.1, respectively).

We used negative binomial generalized linear models to perform a differential analysis of the GIT microbiome OTU counts of *T. bryosalmonae*-positive and -negative fish (Fig. 4; Table S2). These models allow estimation of the differences in dispersion and logarithmic fold changes in GIT OTU counts/abundance between two groups by using the Wald test. *T. bryosalmonae*-infected fish had 10-fold more OTUs (*n* = 202) in the GIT microbiome, and only a small proportion of OTUs (*n* = 19) were more abundant in parasite-free fish. OTUs that strongly increase in number (log_2_ fold change, >3.5) in *T. bryosalmonae*-infected fish mostly belong to nonpathogenic aquatic, anaerobic sediment/sludge, or ruminant bacteria (Table S2). Roughly 50% (*n* = 112) of these OTUs were also detected in water samples; however, only a few of these OTUs (*n* = 4) were abundant in the water samples. The most strongly decreased OTUs in *T. bryosalmonae*-infected fish were close relatives of bacteria of the genus *Yersinia* that are found in the bacterial communities in healthy skin tissues (33) and the gut microbiome of insects.

10.1128/mSphere.00418-17.4 TABLE S2  Most strongly overrepresented OTUs in abundance and their phylogenetic affiliations in the GITs of *T. bryosalmonae*-positive fish and those of parasite-free trout. The last OTU (FN908458) was less abundant in the GITs of *T. bryosalmonae*-positive fish than in those of parasite-free fish. padj, adjusted *P* value. Download TABLE S2, DOC file, 0.1 MB. Copyright © 2017 Vasemägi et al. 2017 Vasemägi et al. This content is distributed under the terms of the Creative Commons Attribution 4.0 International license.

## DISCUSSION

The GIT of fish supports a complex and dynamic microbial ecosystem that is intimately linked to host nutrient acquisition, epithelial development, immune system priming, and disease prevention. Earlier work has shown that the fish GIT microbiome is influenced by size, developmental stage, season, and diet (34–40); however, the effects of and interactions among GIT bacterial communities, pathogens, and parasites remain largely understudied. Here, we evaluated for the first time how the microbial richness within the GITs of juvenile salmonid fish is linked to an endemic parasitic disease. Second, we compared the microbial communities within the GITs of young-of-the-year (0+) brown trout and river water to shed light on the colonization and origin of GIT microbiomes. In addition, to further understand the interplay among abiotic parameters of the habitat, host condition, and disease status, we tested whether various ecological environmental factors, host population genetic divergence, and gender associate with the GIT microbiome of brown trout. Altogether, our results demonstrate the importance of multiple abiotic and biotic factors influencing GIT microbiomes in natural fish populations.

On the basis of earlier work, we hypothesized that the microbial diversity within the GITs of brown trout will show considerable intra- and interpopulation variations and will be strongly linked to the ecological status/geomorphology and water temperature of the river they inhabit. Indeed, we found that the microbial composition has a high degree of interindividual variability, even within populations, and strong clustering according to the population of origin. This corroborates earlier studies of wild guppies (41), sticklebacks (42), and Atlantic salmon (36). In contrast, the microbial community in river water showed less within-river variation but, like the GIT community, also showed strong separation according to the river of origin. Consistent with earlier studies, only a very small number of OTUs (*n* = 14) were present in a majority of the gut samples, representing a “core community” (41, 42). Similarly, the number of OTUs in the “core community” within water samples was low, with only 12 common OTUs found in >50% of the water samples. The latter observation can be partly explained by our sampling strategy, which included only a single time point. Likewise, only a small proportion (11%) of the OTUs were found in both the GITs of trout and river water. This is in accordance with earlier studies that reported highly dissimilar microbe compositions in fish guts and water.

As reviewed in reference 43, studies on lotic systems have indicated that OTUs in the free-living stream are affiliated with *Actinobacteria*, *Bacteroidetes*, and *Proteobacteria* (Fig. 1, second panel from the top). We found three prominent OTUs that are highly abundant in all of the streams we studied, an unidentified member of the family *Microbacteriaceae* (closest match, AJ575497), a *Flavobacterium* species (EU801584), and *Limnohabitans* species (JF697493 and GQ340121) (Fig. 1, bottom). The *Microbacteriaceae* OTU belongs to one of the dominant freshwater actinobacterial clusters, acII (44). The *Limnohabitans* species, especially those affiliated with the R-BT lineage, are known to inhabit a broad range of freshwater habitats (45). The genus *Flavobacterium* is rich in species found in a wide spectrum of habitats (46), they are abundant in freshwater and brackish water environments (47–50), and the most abundant *Flavobacterium* OTU in our study was most similar to a free-living brackish water phylotype from the Chesapeake Bay (51). These observations corroborate that the dominant bacteria in the streams are typical ubiquitous freshwater organisms.

Typical phyla found in the GIT communities belong to *Proteobacteria*, *Firmicutes*, *Actinobacteria*, and *Chloroflexi* (Fig. 1, second panel from the top); however, few OTUs are shared by phyla abundant in both stream water and GIT communities. For example, the OTUs found in both *Actinobacteria* and *Proteobacteria* are not the same (Fig. S2). Earlier studies of the *Salmo salar* GIT microbiome identified *Firmicutes*, *Bacteriodetes*, and *Actinobacteria* as common phyla in smolts and parr (36). That same study found that OTUs in the core microbiota of freshwater parr are commonly assigned to the genus *Yersinia* and several other members of the family *Enterobacteriaceae*. Our observations confirm that OTUs from *Yersinia* are common, yet we also find that other gut enterobacteria, such as *Shigella*, and *Serratia*, dominate in the guts of salmonid parr (Fig. 1, third panel from the top). In a large variety of host organisms, a high abundance of *Clostridiales*, *Bacilli*, *Lactobacilli*, and *Corynebacteriales* bacteria in the GIT has been reported (52–54). A characteristic common to all of these groups is a preference for an anaerobic environment such as that found in animal intestines. It is therefore likely that most of the bacteria found in fish guts, even in those of juvenile fish, represent commensals and symbionts rather than a passive collection of environmental bacteria (55).

The microbial community in lotic systems has been shown to depend on various factors, such as the concentration of metals, the temperature, the quantity and quality of organic matter, and hydrological factors (43). Among abiotic environmental factors, temperature is among the most important for poikilothermic organisms because it governs physical, chemical, and biological reactions. Temperature also affects the immune responses, metabolic rates, enzyme activities, digestion rates, and somatic growth of fish (56–58). Moreover, temperature, oxygen concentration, and pollutants may act as environmental stressors and have an important impact on the microbial community in the gut because of a weakening of the host’s immune system (34). Therefore, we hypothesized that multiple environmental factors may covary with variation in the microbial composition between rivers. Consistent with this, the bacterial composition in the GITs of brown trout showed significant associations with water temperature, oxygen conditions, and river geomorphology (i.e., the size of the catchment area, river length, and the number of dams in the upstream water course). This indicates that both the habitat and abiotic environmental factors are important drivers of microbiome differentiation in the fish gut (41, 42).

Similar to an earlier report (59), both the length and the mass of fish showed significant relationships with the GIT microbiome. However, the GIT microbiota in juvenile trout was not affected by gender, which contrasts with an earlier report on perch and sticklebacks (38). However, it is possible that the strong interpopulation divergence of GIT microbiomes overshadows relatively weak effects of gender, as typically observed in vertebrates (42, 59, 60). Similarly, we did not observe significant associations between the genetic diversity/divergence of the host and the bacterial diversity/divergence within the GIT. This suggests that processes resembling random genetic drift do not explain variation in the GIT community structure in juvenile brown trout. This finding contrasts with recent work by Smith et al. (42), who reported a positive correlation between fish population genetic distance and gut microbiota distance in three-spined sticklebacks. However, because their work was based on a small number of populations (*n* = 6), the question of whether genetic divergence in geographically close fish populations is related to the divergence in the gut microbiota remains unsolved. Thus, more studies are needed to characterize the relationships among different environmental, physiological, genetic, and evolutionary factors that influence the microbial communities within the fish gut.

Because the gut represents a key habitat for dynamic interactions among the host, microbes, and components of its environment, we hypothesized that *T. bryosalmonae*-infected juvenile trout have less diversity/richness of bacteria and are colonized by more opportunistic/pathogenic bacteria. However, we did not find any evidence of decreased diversity of GIT microbiomes in relation to disease and instead found that the richness of the GIT microbial community increased with the parasite load (Fig. S2). Thus, parasite infection does not necessarily lead to reduced diversity in the GIT microbiome, as shown for homologous pathogens such as *Aeromonas salmonicida* (31). In addition, instead of an excess of opportunistic/pathogenic bacteria, we observed that the GIT communities of *T. bryosalmonae*-infected trout are overrepresented by OTUs that most likely originated in the surrounding river water community. This may be a sign of the weaker homeostasis of infected fish because we observed a higher degree of colonization (transient environmental effects). Thus, our results contrast with those of an earlier report (http://afs-fhs.org/perch/resources/14069237543.2.7pkd2014.pdf) that suggested that increased PKD mortality rates are associated with secondary infection with *A. salmonicida* or *Flexibacter columnaris* or with the disease ichthyophthirius multifilis. Alternatively, given that our samples were collected late in September, it is still possible that PKD has a more drastic effect on the microbiome in the host GIT during summer months, when the water temperature is higher and juvenile fish are more vulnerable to stressors. Therefore, it would be interesting to evaluate the relationships between host microbial communities and PKD at high water temperatures.

Here we present novel evidence about interactions of the intestinal microbiome in relation to parasitic disease, environmental factors, and natural fish population structure and physiology. The diversity among bacteria in the GIT corroborated previous observations at a high taxonomic level (phyla) but differed at the genus and OTU levels. The heterogeneity at the genus and OTU levels was high within and between fish populations, but the bacteria were found to be strictly composed of commensals/symbionts in the GITs of young fish. PKD had severe effects on host physiological processes, and *T. bryosalmonae* infection affected the composition of the microbiota of the GITs of juvenile brown trout, directing it toward weakened homeostasis. Our results underscore the importance of the complex interactions among parasites, symbiotic gut bacteria, and the physiological condition of the host. This information will help us to understand the selective pressures governing microbial community assembly and relationships among host fitness, its microbial composition, and disease etiology.

## MATERIALS AND METHODS

### Collection of fish and water samples and environmental variables.

**FIG 5:**
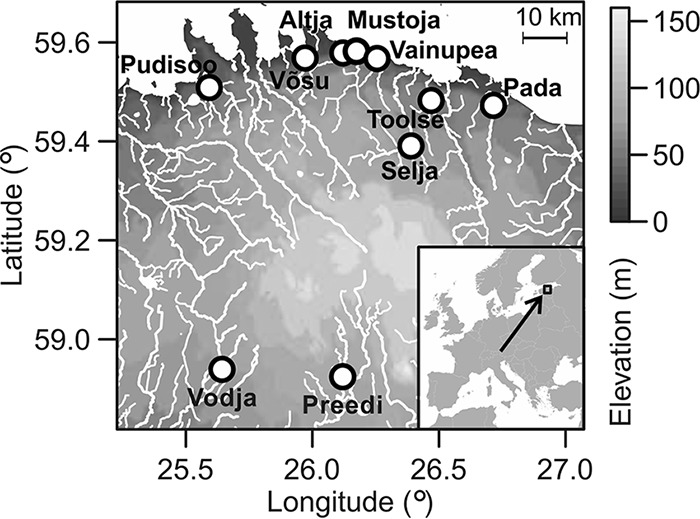
Geographic location of the region. Sampling positions on the 10 rivers are represented by white circles.

All samples were collected from 11 to 14 September 2013 from 10 rivers that drain into the Baltic Sea (Fig. 5). Juvenile trout were collected from 10 genetically distinct but geographically close populations, as the primary aim of our study was to evaluate the effects of parasite and disease severity on the GIT microbiota (61). In total, 105 of the 0+ brown trout collected (7 to 17 per river; Table S3) were caught by electrofishing in 50- to 100-m river stretches (fishing permit 82/2013, Estonian Ministry of Environment). Fish were euthanized immediately after being caught, the whole mid and distal intestine (from the anus to the pyloric cecum) was dissected with a sterile scalpel and forceps, and samples were stored at +4°C. Triplicate 1-liter water samples were collected from all of the rivers immediately upstream of the fish collection sites, transported (kept at 4°C) to the laboratory within 2 to 8 h, filtered with 0.22-µm polycarbonate filters (47-mm diameter; Poretics), and stored at −20°C. Replicate samples were not filtered in subsequent order but were mixed to avoid longer storage of some river water samples than others at 4°C.

10.1128/mSphere.00418-17.5 TABLE S3  Abundances of PKD-infected and noninfected fish. Download TABLE S3, DOC file, 0.04 MB. Copyright © 2017 Vasemägi et al. 2017 Vasemägi et al. This content is distributed under the terms of the Creative Commons Attribution 4.0 International license.

Data regarding river length, the size of the catchment area, the number of dams, and the total area of reservoirs upstream of the sampling sites were obtained from the Estonian Nature Information System (EELIS, http://loodus.keskkonnainfo.ee/eelis/) electronic database. The oxygen concentration and water temperature at the time of sampling were measured with a Marvet junior oxygen meter (Elke Sensor OÜ) and HOBO 8K Pendant temperature/alarm data loggers (Onset Computer Corporation). In addition, we included two river ecological status indices in our subsequent analyses, the trophic diatom index (TDI) (62) and the numerical environmental quality ratio (EQR) based on zoobethos species diversity (63).

### Bacterial DNA isolation, amplification, barcoding, and DNA sequencing.

Each intestinal tract was dried of excess ethanol on a tissue and cut linearly along the bowel. The intestinal content was scraped off with a small, round-edged, sterile spoon and put into a tube with 1× Tris-EDTA (pH 7.4) and 0.5% SDS. A half gram of zirconium powder was added to the solution, homogenized with MP Fast Prep-24 for 40 s (speed, 6 ms^−1^), and centrifuged for 1 min at 9,500 × *g*. The supernatant was placed into a new tube and gently mixed with 900 µl of a lysis solution (5.25 M guanidine thiocyanate [GuSCN], 0.1 M Tris-HCl, 0.2 M EDTA [pH 6.4]). A vortexed silica suspension was added to the previous mixture, which was incubated for 5 min at room temperature and then centrifuged for 30 s at 2,400 × *g*, and the supernatant was discarded. One milliliter of 5 M GuSCN was gently mixed with the precipitate, and the mixture was centrifuged for 30 s at 2,400 × *g*. One milliliter of 50% ethanol was then added, and the mixture was centrifuged at 7,400 × *g* for 30 s. The precipitate was dried for ~45 min at 40°C and diluted in 100 µl of DNA- and nuclease-free H_2_O and incubated for 10 min in 37°C. After that, the solution was centrifuged for 1 min at 16,000 × *g* and the supernatant was transferred to a new tube.

For Illumina sequencing (PE250) of amplicons, the DNA (including negative DNA extraction and a nontemplate control) was amplified with nonbarcoded 16S rRNA gene primers that target the V3-V4 hypervariable region (64). DNA was amplified (in a total volume of 20 µl) as follows: 1× Physion Mastermix, 0.75 µg·ml^−1^ bovine serum albumin, 10 µM both 16S rRNA bacterial primers, 10 µg of template DNA, and PCR grade double-distilled H_2_O (ddH_2_O). The cycling conditions were 30 s at 98°C; 27 cycles of 98°C for 10 s, 55°C for 30 s, and 72°C for 15 s; and 72°C for 10 min. For the barcoding stage, all reaction mixtures contained 1× Physion Mastermix, 0.8 µM multiplex solution, 0.2 µM P5/P7 index, 1 µl of the first-stage PCR product diluted 1:20, and ddH_2_O in a total volume of 20 µl. The cycling conditions for the second stage comprised 2 min at 98°C; 12 cycles of 98°C for 20 s, 60°C for 30 s, and 72°C for 30 s; and 72°C for 5 min. Amplification products were purified and sequenced with MiSeq (Illumina PE250) at the Finnish Institute of Medical Microbiology.

### Quantification of relative parasite loads.

DNA extraction and quantification of *T. bryosalmonae* within the kidney were performed by comparing the amounts of parasite DNA and host DNA by quantitative PCR as described by Bruneaux et al. (17).

### Genetic diversity within and divergence among populations and molecular identification of gender.

Genotype data for nine microsatellite loci were obtained from previously published work by Koljonen et al. (65), Ozerov et al. (66) and Debes et al. (18). Calibration of the microsatellite alleles was carried out by comparing the alleles genotyped for the same population. The genetic diversity within each population was quantified by calculating allelic richness (*A*_r_), which takes into account the uneven sample size. The genetic divergence between populations (FST) was calculated as described by Weir and Cockerham (67). Isolation by distance between FST and geographic distances was done with the Mantel test. The molecular gender of fish was determined by amplifying a male-specific 148-bp SDY fragment as described by Aykanat et al. (68), by using 2% agarose electrophoresis to visualize the male-specific fragment.

### Sequence analysis.

The total pool of sequences (2,701,997; quality filtered with Trimmomatic v 0.32 [>Q30]) obtained from demultiplexed MiSeq reads was clustered (at 97% similarity within the V3-V4 regions of 16S rRNA gene sequences) into 6,796 nonchimeric OTUs with cd-hit-otu (69) and affiliated by using the SILVA database (ver 115) with SINA aligner (70). Absolute counts were used to analyze alpha diversity and test for variation between biological replicates. After the removal of OTUs found in fewer than nine samples, 3,489 OTUs remained in the data set. These were the data used to analyze beta diversity.

### Statistical analysis.

To test for variation within river water triplicate samples and within fish specimens from the same river, we used an ANOVA-like permutation test in the R extension vegan (71, 72). When calculating pseudo-*F* values, type III effects were taken into account. To estimate the extrapolated species richness in a species pool, the ACE index and Shannon diversity index were calculated by using R (vegan).

We used an analysis of variance using distance matrices known as PERMANOVA (73) to test the range of variation in OTU diversity in different rivers. dbRDA was used (74) with the vegan package to analyze the relationship between OTU diversity and explanatory variables such as the morphometry and environmental parameters of the rivers and fish morphometry and physiology traits. To calculate the dissimilarity matrix, a Bray-Curtis distance-based transformation was used.

A nonparametric Mantel test was used to test for nonrandom associations between the geographic distance between sampling sites and the dissimilarity of the GIT microbiome (Bray-Curtis distance) between populations. We also used the Mantel test to evaluate the association between the geographic distance and genetic divergence between fish populations (isolation by distance). To test for a correlation between the host population genetic diversity (*A*_r_) and the GIT microbiome (Shannon) diversity index, we used the Spearman rank correlation coefficient.

Differential-abundance comparisons of parasite-infected and noninfected fish were made by using a negative binomial Wald test (75). To model the dependence of GIT richness on the parasite load, the ACE index was modeled by using mixed linear models (R [lme4]) and using the origin of fish (river) as a fixed factor, the square-root-transformed relative amount of parasite DNA in fish blood as a covariate, and the biological replicate as a random factor.

### Accession number(s).

All of the sequences obtained in this study have been deposited in the NCBI Sequence Read Archive under BioProject PRJNA388139.
