# Supplementary figures and images for: Effect of Environmental Factors and an Emerging Parasitic Disease on Gut Microbiome of Wild Salmonid Fish
Source: mSphere. 2017 Dec 20;2(6):e00418-17. doi: 10.1128/mSphere.00418-17 (PMC5737052; doi:10.1128/mSphere.00418-17)

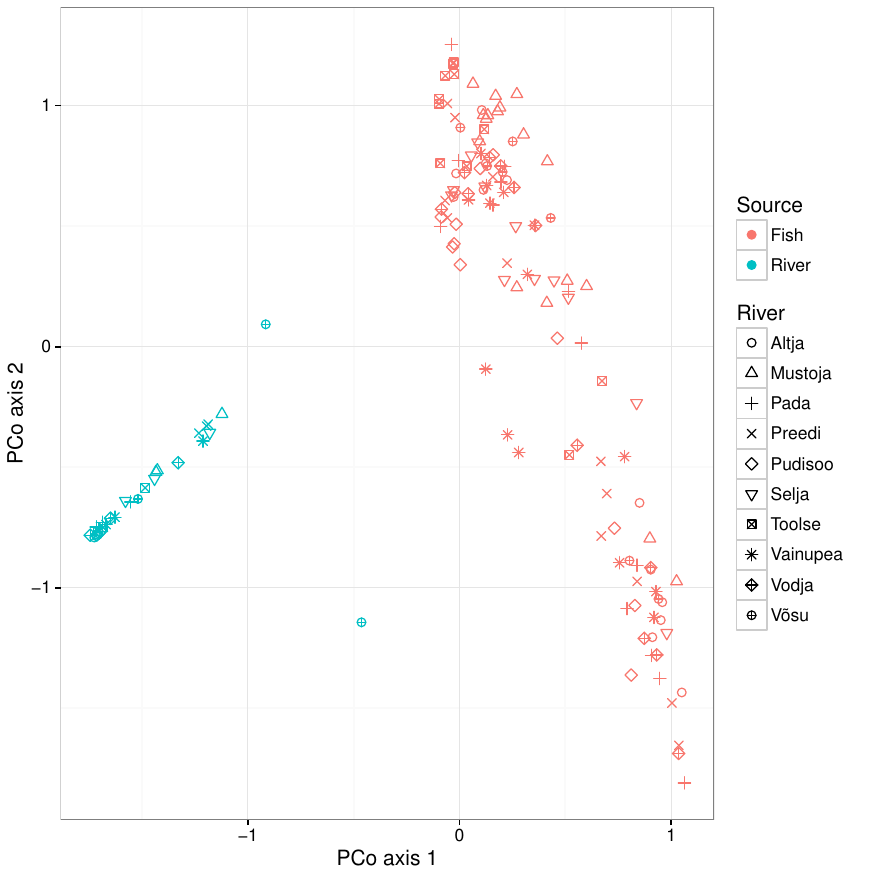

Supplement: FIG S1 [file sph006172430sf1.tif]

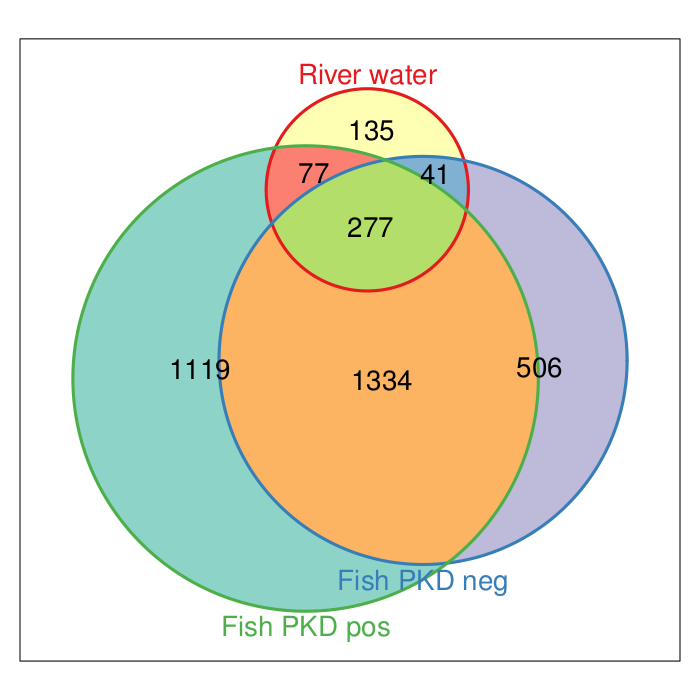

Supplement: FIG S2 [file sph006172430sf2.tif]

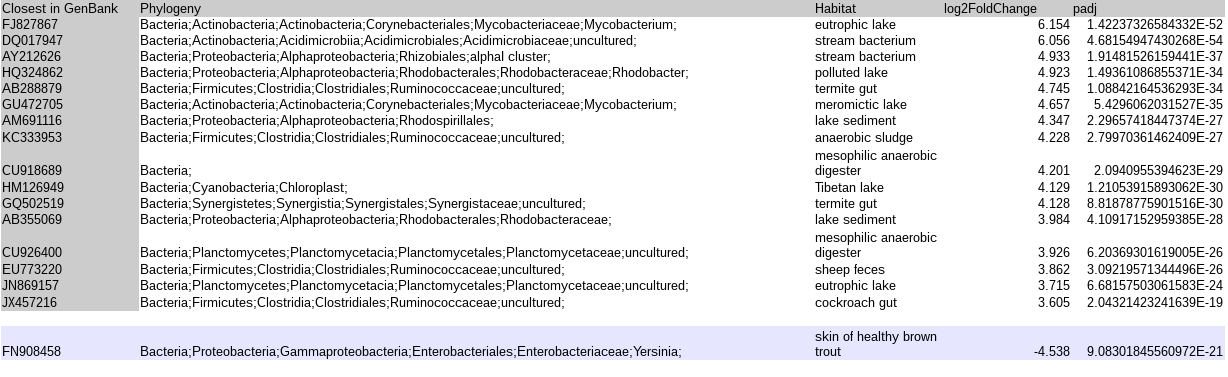

Supplement: TABLE S2 [file sph006172430st2.doc]
