# Supplementary material for: Effect of Environmental Factors and an Emerging Parasitic Disease on Gut Microbiome of Wild Salmonid Fish
Source: mSphere. 2017 Dec 20;2(6):e00418-17. doi: 10.1128/mSphere.00418-17 (PMC5737052; doi:10.1128/mSphere.00418-17)
Supplement: TABLE S1 [file sph006172430st1.doc]

| **River name** | **Length (km)** | **Catchment area (km^2^)** | **Number of dams** | **Dam(s) surface area (ha)** | **100-TDI^*^** | **EQR-zoobentos^**^** |
| --- | --- | --- | --- | --- | --- | --- |
| Altja | 17.6 | 46.1 | 1 | 3.5 | 39 | 0.92 |
| Mustoja | 28.0 | 138.9 | 3 | 13.0 | 41.6 | 0.96 |
| Pada | 40.5 | 191.1 | 1 | 0.2 | 12 | 0.68 |
| Preedi | 27.1 | 291.5 | - | - | 65.1 | 0.84 |
| Pudisoo/ Pärlijõgi | 31.8 | 143.7 | 2 | 1.2 | 48 | 0.84 |
| Selja | 47.6 | 422.6 | 2 | 4.0 | 30.8 | 0.92 |
| Toolse | 25.2 | 84.3 | - | - | 29.6 | 0.96 |
| Vainupea | 27.4 | 55.6 | 1 | 0.6 | 39 | 1.00 |
| Vodja | 18.8 | 79.7 | - | - | 48.1 | 0.80 |
| Võsu | 24.7 | 63.6 | 4 | 18.9 | 31 | 0.92 |

* -TDI - Trophic Diatom Index

** - EQR - Environmental Quality Ratio
