# Supplementary material for: Effect of Environmental Factors and an Emerging Parasitic Disease on Gut Microbiome of Wild Salmonid Fish
Source: mSphere. 2017 Dec 20;2(6):e00418-17. doi: 10.1128/mSphere.00418-17 (PMC5737052; doi:10.1128/mSphere.00418-17)
Supplement: TABLE S3 [file sph006172430st3.doc]

| **River name** | **Infection status** | **N** |
| --- | --- | --- |
| Altja | positive | 12 |
|  | negative | 0 |
| Mustoja | positive | 17 |
|  | negative | 0 |
| Pada | positive | 0 |
|  | negative | 13 |
| Preedi | positive | 0 |
|  | negative | 12 |
| Pudisoo | negative | 6 |
|  | positive | 6 |
| Selja | negative | 2 |
|  | positive | 10 |
| Toolse | positive | 0 |
|  | negative | 11 |
| Vainupea | negative | 11 |
|  | positive | 3 |
| Vodja | positive | 0 |
|  | negative | 12 |
| Võsu | positive | 7 |
|  | negative | 0 |
